# Spatio-temporal patterns of proportions of influenza B cases

**DOI:** 10.1038/srep40085

**Published:** 2017-01-09

**Authors:** Daihai He, Alice P. Y. Chiu, Qianying Lin, Duo Yu

**Affiliations:** 1 Department of Applied Mathematics, Hong Kong Polytechnic University, Hung Hom, Kowloon, Hong Kong (SAR) China; 2 Department of Biostatistics, School of Public Health, University of Texas Health Science Center at Houston, United States

## Abstract

We studied the spatio-temporal patterns of the proportions of influenza B cases out of all typed cases, with data from 139 countries and regions downloaded from the FluNet compiled by the World Health Organization, from January 2006 to October 2015. We restricted our analysis to 34 countries that reported more than 2,000 confirmations for each of types A and B over the study period. Globally, we found that Pearson’s correlation is greater than 0.6 between effective distance from Mexico and the proportions of influenza B cases among the countries during the post-pandemic era (i.e. Week 1, 2010 to Week 40, 2015). Locally, in the United States, the proportions of influenza B cases in the pre-pandemic period (2003–2008) negatively correlated with that in the post-pandemic era (2010–2015) at the regional level. Our study limitations are the country-level variations in both surveillance methods and testing policies. The proportions of influenza B cases displayed wide variations over the study period. Our findings suggest that the 2009 influenza pandemic has an evident impact on the relative burden of the two influenza types. Future studies should examine whether there are other additional factors. This study has potential implications in prioritizing public health control measures.

Globally, influenza is an important cause of morbidity, mortality and hospitalization. Two major types circulate among human population, type A and type B, and they share many common morphological and epidemiological characteristics. They also have similar clinical presentations^1^. However, they differ in the animal reservoirs that they reside in, influenza A virus infects mainly the mammalian species and birds, while influenza B virus infects only humans and seals. Influenza B virus displays less antigenic drift (at slower rate) and can cause significant epidemics but not pandemic, unlike influenza A^2^. Also, while influenza A have a large number of subtypes^3^, influenza B only diverges into two lineages in the 1970s. These viruses are known as Victoria and Yamagata lineages, which are named after their first representatives B/Victoria/2/87 and B/Yamagata/16/88^4^,^5^.

Previous studies have investigated a trend and burden of influenza B^6^,^7^,^8^. Glezen *et al*. suggested that there was an increasing trend of the proportion of influenza B in both the United States (US) and Europe between 1994 and 2011^9^. Other studies have also explored the potential roles of air travel on the global and regional spread of influenza^10^,^11^,^12^,^13^. These studies showed that air travel volumes and flight distance are important factors driving the spread of influenza^10^,^11^,^12^,^13^. However, to our knowledge, there is a lack of recent literature that examined the global patterns of the proportions of influenza B cases after the 2009 influenza pandemic.

The aim of our study is to examine the spatio-temporal patterns of the proportion of influenza B in the post-pandemic era at both global and regional level. We focus on weekly laboratory confirmations from 74 countries/regions that have the most laboratory-confirmations of influenza A and influenza B from 2006 to 2015, excluding the year of 2009 to avoid the impact of widely differed testing efforts (i.e. testing policies or testing practices) among countries during the 2009 A(H1N1) influenza pandemic, which was originated from Mexico^14^. We define the proportion of influenza B cases as the number of laboratory-confirmations of influenza B out of the total of influenza types A and B confirmations over the whole study period. This measure reflects the relative health care burden of influenza B out of both influenza A and B cases. We focused on two research hypotheses:

1. There is a linear association (i.e. a statistically significant linear relationship as defined by Pearson’s correlation coefficient) between the proportion of influenza B and effective distance from Mexico, given that the pandemic H1N1 strain spread from Mexico and Northern America to the rest of the world. Simonsen *et al*.^15^ found “far greater pandemic severity in the Americas than in Australia, New Zealand, and Europe” in 2009. He *et al*.^16^ found that the H1N1pdm (pandemic) strain skipped a large part of Europe and East Asia but not in the Northen America in the 2011/12 influenza season.
2. In the US, there is a linear association between the pre-pandemic and post-pandemic era at the regional level, given that influenza B and influenza A (especially the H3N2 strain) showed anti-phase patterns. Namely, when influenza A/H3N2 are severe, the other strains are mild.

## Data Collection

Influenza data are downloaded from the FluNet, a publicly available and real-time global database for influenza virological surveillance compiled by the World Health Organization. Data from FluNet primarily comes from three different sources: They are provided by the National Influenza Centres of the Global Influenza Surveillance and Response System (GISRS), uploaded from WHO regional databases or other national influenze reference laboratories that have active collaborations with GISRS. We obtained raw data from January 2006 to October 2015, covering 138 countries. We excluded data from the year 2009, to avoid the impact of excessive testing during the 2009 A(H1N1) influenza pandemic in many countries. First, we compute the total number of confirmations of influenza A and influenza B respectively across the time period. Second, we compute the proportion of influenza B, which is defined as the number of confirmations of influenza B out of the total number of confirmations of influenza A and B. To reduce the impact of statistical noise from countries that provided only a small amount of data, we restricted our analyses at two threshold levels (*θ*), i.e. those countries that provided at least 500 or 2,000 confirmations for each of influenza A and influenza B across the time period. Among those countries that had reached the 500 confirmations threshold, we also obtained data on the number of confirmations by influenza B lineage, i.e. B/Victoria, B/Yamagata or uncharacterised influenza B.

In addition, we downloaded laboratory confirmations for influenza A and influenza B from the Centre of Health Protection in Hong Kong from January 1998 to October 2015 (http://www.chp.gov.hk). Due to data availability and quality, we also downloaded regional level laboratory confirmations of influenza A and influenza B from FluView of the Centers for Disease Control and Prevention in the U.S. from January 1997 to October 2015 (http://www.cdc.gov/flu/weekly/). Again, we computed the proportion of influenza B using the same method for the period January 2006 to October 2015.

We collected information about individual countries including their population size as of 2005^17^, longitudinal and latitudinal information^18^ and flight data from the Official Airline Guide (OAG, http://www.oag.com).

## Methods

For hypothesis 1, we computed the Pearson’s correlation coefficient between the proportion of influenza B and the effective distance from Mexico. Effective distance is a function of the mean daily number of flight passengers and the distance of the shortest flight path between two places^19^. As a comparison, we repeated the analysis using China as the reference country, instead of Mexico. In addition, we also computed the Pearson’s correlation coefficient between the proportion of influenza B and the longitude and latitude in absolute terms. We varied the threshold of influenza specimen at two levels, 500 and 2,000. We also considered different time periods, i.e. pre-pandemic period, post-pandemic period or both periods.

For hypothesis 2, we computed the Pearson’s correlation coefficient between the proportion of influenza B in the pre-pandemic and post-pandemic era at the regional level in the United States. We defined the pre-pandemic period by varying the starting year between 1997 and 2007, and the ending year as 2008. The post-pandemic period was fixed at January 2010 to October 2015. This resulted into nine pairs of regional level data for the pre- and post-pandemic proportion of influenza B. We repeated the computation of the Pearson’s correlation coefficient for each of these starting years.

We performed lineage-level analysis by plotting heat maps to compare the patterns of confirmations of influenza B lineages against effective distance (definition 1) from Mexico, effective distance (definition 2) from Mexico, longitude, latitude and country’s median age.

We constructed five different statistical models to determine how the proportion of influenza B is associated with the other factors. Model 1 is a linear mixed effect model. The response factor is the proportion of influenza B. The independent fixed factors include population size, longitude, absolute latitude and effective distance. Geographic region is an independent random factor.

Model 2 is also a linear mixed effect model, with the same factor as Model 1, except that we use the number of laboratory specimens tested to replace the population size.

Model 3 is a linear model. The response factor is the proportion of influenza B. The independent factors are population size, longitude, absolute latitude and effective distance.

Model 4 is also a linear model. We have the same factors as in Model 3, except that we use the number of laboratory specimens tested to replace the population size.

In addition, we created a linear model (Model 5), with the proportion of influenza B as response factor, and population size, longitude, absolute latitude, effective distance and region as independent factors. We removed the independent factors one at a time, and then re-assessed the model fit by re-running the linear model and computing its Akaike Information Criterion (AIC).

For the US, we also conducted regional level analysis on the correlation between the proportion of influenza B pre- and post-2009 influenza pandemic. We defined the post-pandemic era as the period from January 2010 to August 2015. For the pre-pandemic period, we varied the starting period from 1997 to 2007, while fixing the ending period to be 2008.

All statistical analyses are conducted using statistical package R version 3.2.2. Statistical significance is assessed at 0.05 level. For multiple-testing, we applied the Bonferroni adjustment to the significance level^20^.

## Methods of Computing Effective Distance

We obtained global air traffic data from the Official Airline Guide (OAG, http://www.oag.com). Briefly, the data contains the mean daily number of seats on the flight route between each pair of international airports in 2009. We made a simplifying assumption that the actual number of passengers travelled in these routes are proportional to the number of flight seats. We aggregated airports that are located within the same country into individual nodes, and we defined edges as paths that connect different nodes together. Based on these data, we developed a global air traffic network with 209 nodes and 4,700 edges representing the countries and their flight connections. Myanmar’s international air traffic data in 2009 were incomplete in the OAG dataset.

According to Brockmann and Helbing^19^, the effective distance from node *i* to node *j* (Definition 1) is defined as *D*_*ij*_ = 1 − ln(*P*_*ij*_), where *P*_*ij*_ = *T*_*ij*_/*T*_*i*_ is the relative mobility rate from *i* to *j. T*_*ij*_ represents the number of flight passengers travelling from node *i* to *j*, and 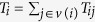 represents the total number of flight passengers from node *i* to all possible destinations within the global air traffic network. We define the distance from node *i* to node *j* as the sum of the length of the edges that connect them, and the effective distance as the shortest of such paths.

The definition of effective distance is arbitrary. We adopted another definition (Definition 2) for comparison purposes. We define *D*_*ij*_ = −ln(*ω*_*ij*_), where *ω*_*ij*_ = *T*_*ij*_/*N*_*i*_ and *N*_*i*_ is the population size of country *i*. There is a strong correlation between the effective distances using Definition 1 and Definition 2(*p*-value < 0.001).

We also compared the correlation between the proportion of influenza B and the effective distance from Mexico using either definition of effective distance, and both displayed a strong correlation.

## Results

### Global Patterns

**Figure 1:**
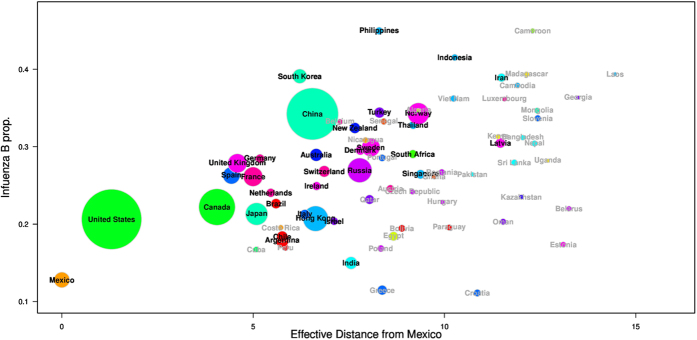
The proportion of influenza B from 72 countries against their effective distance from Mexico (week 1 of 2010 to week 40 of 2015). We use disks to represent individual countries. The size of the disk is proportional to square root of the original size in order to enlarge the smaller values. Names of the top 34 countries used in the main analysis are in black and others in dark grey. Countries are coloured according to their geographic regions or sub-regions.

Figure 1 shows the proportion of influenza B versus the effective distances from 72 localities to Mexico. The proportion of influenza B is significantly and positively correlated with the effective distance, i.e., the further from Mexico the higher the proportion. Pearson’s correlation is 0.617 (95%CI: 0.352, 0.790) and *p*-value is less than 0.001 among the top 34 countries where both types A and B confirmations are more than 2,000. It is also evident that countries in the same geographic region (i.e. as represented by the same colour) tend to have similar influenza B proportions. The correlation became weaker but still significant after we inserted data from November 2015 to February 2016.

As a comparison, if we choose China as the reference country, the correlation of proportion of influenza B versus the effective distance from 32 localities to China is not statistically significant (*p*-value = 0.3733).

**Figure 2:**
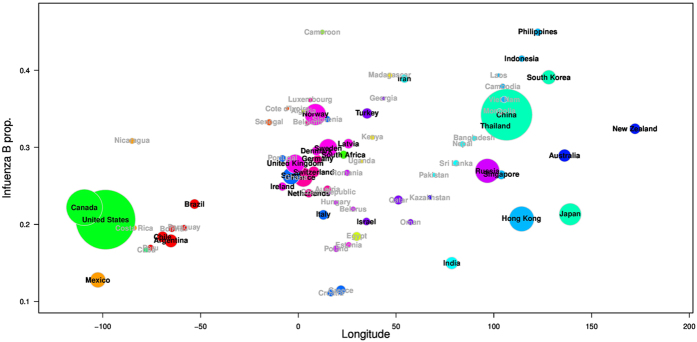
The proportion of influenza B from 74 localities against their longitudes (week 1 of 2010 to week 40 of 2015). We use disks to represent individual countries. The size of the disk is proportional to square root of the total confirmations of influenza A and influenza B. Names of the top 34 countries used in the main analysis are in black and others in darkgrey. Countries are coloured according to their geographic region or sub-region.

Figure 2 shows the proportion of influenza B versus the longitude of each country. Pearson’s correlation is 0.538 (95%CI: 0.244, 0.741) and *p*-value is 0.001 among the top 34 countries where both types A and B confirmations are more than 2,000.

Table 1 shows the results of the Pearson’s correlation coefficients of the effective distance from Mexico or China, longitude and absolute latitude. We also varied the threshold levels at 500 or 2,000, and the time period to include the pre-pandemic period only, post-pandemic period only or both periods. For the effective distance from Mexico, there demonstrated a strong positive correlation of 0.617 (95% CI: 0.352, 0.790) (using definition 1) and 0.626 (95% CI: 0.365, 0.796) (using definition 2) with the proportion of influenza B when we considered a threshold of 2,000 for the entire study period (*p*-value < 0.001 in both cases). There was a weak negative correlation for the effective distance from China but they were not statistically significant (*p*-value = 0.3733). For longitude, significant correlation of 0.538 (95% CI: 0.244, 0.741) was demonstrated (*p*-value = 0.001). For absolute latitude, the correlation was not significant (*p*-value = 0.8173). We applied Bonferroni correction to adjust for multiple testing, since we tested for multiple factors^20^. We use the new critical *p*-value, *α*/*n*, where *α* is the original critical *p*-value (i.e. *α* = 0.05) and n is the number of factors. After this adjustment, the correlations between proportions of influenza B cases and effective distance or longitude in the post-pandemic era were still significant.

**Figure 3:**
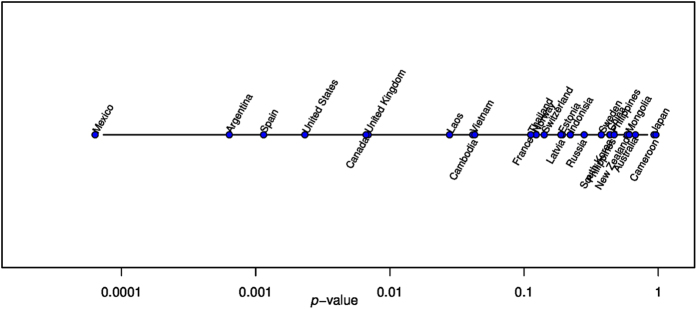
The *p*-values of correlations between influenza B proportion and the effective distance from a reference country.

We show the results with either Mexico or China as the reference country in Table 1. To test the robustness of our findings, we repeat the analyses by shifting the start date to 2011-1 and 2012-1 (i.e. excluding (2009–2010) and (2009–2011)’s data respectively. The results are given in Supplementary Table S1. With a start date of 2011-1, the p-values are only marginally changed. For the start date of 2012-1, the changes are evident but the focal indicators are still significant. Thus, we concluded that the extensive testing policy associated with pandemic influenza in A/H1N1 in 2009 and early 2010 has little impact on our findings. We also conducted sensitivity analysis by using other countries that have either large number of influenza confirmations or high proportion of influenza B as the reference country, and then we repeated the computation of linear association between proportion of influenza B and the effective distance from the reference country. Figure 3 shows the *p*-values for 27 countries. It can be seen that Mexico attains the smallest *p*-value. Interestingly, the other two Spanish-speaking countries, Argentina and Spain, also have relatively small *p*-values when they are the reference countries.

In Supplementary Materials S3, we display the heatmaps of the patterns of B/Victoria, B/Yamagata and influenza B with uncharacterised lineage against definition 1 of effective distance (Supplementary Figure S1), definition 2 of effective distance (Supplementary Figure S2), median age (Supplementary Figure S3), longitude (Supplementary Figure S6) and latitude (Supplementary Figure S7). Consistently across these five figures, we found that influenza B with uncharacterised lineage constitutes a larger number than B/Victoria or B/Yamagata lineages, as reflected by their darker shades. Apart from that, we do not see any other patterns.

In Supplementary Results S5, our statistical models (Models 1–4) also demonstrated consistent results. In Model 1, effective distance was the strongest predictor while longitude was the second strongest predictor. In Models 2 to 4, longitude was the strongest predictor, while effective distance was the second strongest predictor. In Model 5, we assessed the effect of single term deletions from a full linear model. Among the five independent factors being studied, i.e. population size, longitude, absolute latitude, effective distance and region, we found that effective distance (Mexico) shows the strongest improvement. Here the smaller the absolute value of AICc, the better the model is. The results are consistent with the other models, that the effective distance is the most important factor.

The significantly positive correlation we found here continued to hold is restricting to the post-pandemic era (2010–2015), and exclude the pre-pandemic era (2006–2008). However, the pre-pandemic era (2006–2008) alone is not statistically significant.

Note that the global surveillance effort in the post-pandemic era, both in terms of the number of specimens processed in most countries and the numbers of countries reporting data to FluNet, has been improved after the 2009 pandemic. In the United States, where surveillance has been relatively consistent starting from 1997, we found an interesting negative correlation of the proportion of influenza B in the post 2009 pandemic era versus the pre-2009 pandemic period. The results are described in the following section.

### Regional patterns in the United States

**Figure 4:**
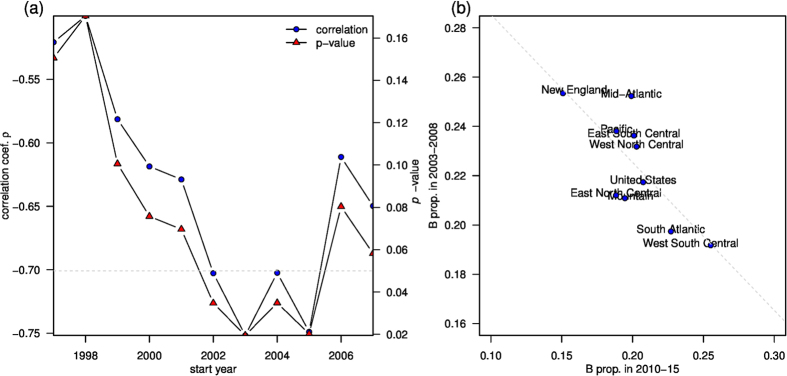
The proportion of influenza B in the pre-pandemic period negatively correlates with that of the post-pandemic era among nine census regions of the United States. The post-pandemic era is defined from Jan 2010 to August 2015. We vary the start date of the pre-pandemic from 1997 to 2007, and fix the end of the pre-pandemic era to be 2008. It is clear that there is a window period such that the proportion of influenza B shows a significantly negative correlation between pre- and post-pandemic periods. (**a**) Correlation and *p*-value as a function of start year of the pre-pandemic period. The *x*-axis shows the start of the pre-pandemic period. (**b**) The correlation between 2003–2008 versus 2010–2015.

Figure 4 shows the correlation of the proportion of influenza B in the pre-pandemic period versus that in the post-pandemic era among nine census regions of the US. We defined the post-pandemic era as from Jan 2010 to August 2015. We varied the start of the pre-pandemic from 1997 to 2007, while we fixed the end of the pre-pandemic era to be 2008. It is clear that there is a window period such that the proportion of influenza B shows a significant negative correlation between pre- and post-pandemic era, and results are shown in Table 2. The Pearson’s correlation between the influenza B proportions in pre-pandemic (2003–2008) and those in post-pandemic (2010–2015) is −0.752, 95% CI is (−0.944, −0.175), and the *p*-value is 0.0195.

## Discussion

To our knowledge, this is the first study on the spatio-temporal patterns of the proportion of influenza B after the 2009 influenza pandemic, at both global and regional levels. We found an evidently positive correlation between the proportion of influenza B and effective flight distance. We also found a significantly negative correlation in the proportion of influenza B between pre- and post-pandemic periods at the regional level in the US.

For the global patterns, we found that the proportion of influenza B significantly correlated with the effective distance from Mexico, with a *p*-value < 0.001. This correlation is robust to the threshold of 500 cases. With higher threshold of 2,000 cases, the correlation is still statistically significant and the correlation coefficient increased. We assess the stability of surveillance by further excluding the post-pandemic data from 2010 and 2011. Our results still remain robust.

Figure 2 shows that the proportion of influenza B also positively correlated with the longitude of countries (Pearson’s correlation = 0.538, (95% CI: 0.244, 0.741), *p*-value = 0.001). Given that the world takes a global shape, where longitude of −180 ≡ +180, longitude is not a very suitable factor. However, these differences among Northern America, Europe and Asia are evident and striking.

We did not find any obvious differences between the patterns of confirmations of B/Victoria and B/Yamagata. This is because influenza B data from FluNet lacked the lineage-level information to allow for a more detailed analysis.

Tafalla *et al*. reviewed the epidemiology of the lineages of influenza B from 2002 to 2013 in nine European countries. They concluded that the lineage circulation showed unpredictable patterns. Also, even after considering neighboring European countries, they were not able to see any common lineage circulation patterns^21^.

To our knowledge, patterns found in Figs 1 and 2 have not been studied before. However, the patterns observed are in line with previous studies^15^,^22^,^23^,^24^, which reported different severity of influenza A (H1N1pdm) or influenza B between Europe and North America. In our study, we considered both 2,000 and 500 confirmations as thresholds. We are interested in the overall proportion of influenza B in each country in the post pandemic era rather than the year to year variations that are subject to stochasticity as well as potential serial correlation across different years due to the slow evolutionary rates of influenza B lineages. By investigating the overall proportion, we have also avoided the differences between Northern Hemisphere and Southern Hemisphere where influenza peaks show a half-year time lag.

Previous studies focused on the impacts of latitude on influenza B patterns. Baumgartner *et al*. studied the influenza activity worldwide and found its seasonality, timing of influenza epidemic period displayed unique patterns according to the latitudinal gradient^25^. Yu *et al*. studied the relationship between latitudinal gradient and the proportion of influenza B among the provinces in China. They found an increasing prevalence of influenza B towards the South^26^. Since influenza virus transmission varies with climatic factors such as temperature and humidity^27^, these influenza B patterns are likely to be explained by climatic factors that vary with the latitudinal gradients. Finkleman *et al*. analysed the patterns of relationship between epidemic patterns of the seasonal activity of influenza A(H3N2), A(H1N1) and influenza B with the country’s latitudinal gradients. They found a positive relationship between latitude and the mean week of epidemic for A(H3N2) and A(H1N1) but not for influenza B^28^. However, at the global level, we did not find any evidence supporting the association with latitude. Caini *et al*. conducted a global study on influenza B which involved 26 countries. They found that the median proportion of influenza B from 2000 to 2013 was 21.4% in the Northern Hemisphere, 24.3% in the intertropical belt and 17.8% in the Southern Hemisphere. There were only borderline significance in the comparison between Southern Hemisphere and the intertropical belt, and no significant differences for the other comparisons^29^. But we observed an interesting relationship between the proportion of influenza B and longitude, which could be associated with the initial patterns of spread of H1N1pdm.

We found that the proportion of influenza B in the period we considered is the lowest among the countries in the South America, followed by North America, Western Europe, Eastern Europe, Middle East and are the highest in Asia. We can see that European countries had a higher proportion of influenza B than United States. It is worthwhile to note that influenza B viruses are more likely to be reported from children^30^. Higher influenza vaccination coverage rate among the general population, and in particular, among healthy school children in the US and Canada, is likely to decrease the proportion of influenza B in these two countries^16^. It will be worthwhile to conduct an in-depth study on these relationships in the future.

Our study found that there is a negative correlation between the proportion of influenza B and pre- and post-pandemic periods in the US. This could be due to cross-reactive immunity as suggested by Ahmed *et al*. where the A(H1N1) pandemic influenza resulted in a large number of persons exposed to this strain in certain countries could have resulted in anti-HA immunity^31^. Whether this phenomenon is also applicable to influenza B virus is worthy of further investigation.

The major strength of our study is our use of longitude and effective flight distance in the study of the proportion of influenza B that are both novel and epidemiologically relevant. Second, the proportion of influenza B is a more robust indicator of the severity of influenza B in a country than the total number of influenza B confirmations or influenza B positive rates (i.e. cases of influenza B out of all influenza specimens).

Our study does have some limitations. First, while the FluNet data permits extraction of global influenza data, those data are subjected to biases, e.g. data could be sporadically entered and testing samples could come from children’s hospital or surveillance testing sites and might not be representative of the general population. The methods of surveillance data collection and their testing policies differ widely across different countries. The intensity of surveillance could change over time. Second, since FluNet’s data does not contain age information of the laboratory specimen, we could not perform further sensitivity analyses of the proportion of influenza B among children or elderly in these countries. Third, FluNet has very limited data on B/Yamagata and B/Victoria, therefore we could not perform a comprehensive analyses of the lineage-specific effects. Fourth, the global air traffic data in 2009 was used for our analysis and comparison with potential changes after the pandemic in 2009. This relies on the strong assumption that air traffic is stable over time. Our results should be interpreted with caution.

## Conclusions

In this study, we have examined the spatio-temporal patterns of the proportion of influenza B globally and in the US. The impacts of different surveillance policies would have been reduced to some extent due to the use of proportion rather than other absolute numbers.

Our results showed that there were wide variations in the proportions of influenza B over the study period. Differences in the proportion of influenza B between Europe and Northern America (i.e. the US and Canada) could be associated with the different influenza vaccination policies and coverage among healthy school children and the general population. The patterns of spread of H1N1pdm could be one of the major reasons. Future studies could examine whether other additional factors, e.g. health access rates, population age structure and climatic factors, which could contribute to the patterns of influenza B proportions. To identify the major factors for prioritizing public health control measures will be both challenging and worthwhile. This study is of both scientific and public health significance.

## Additional Information

**How to cite this article:** He, D. *et al*. Spatio-temporal patterns of proportions of influenza B cases. *Sci. Rep.* **7**, 40085; doi: 10.1038/srep40085 (2017).

**Publisher's note:** Springer Nature remains neutral with regard to jurisdictional claims in published maps and institutional affiliations.

## Supplementary Material

Supplementary Information

## Figures and Tables

**Table 1:** Summary of all Pearson’s correlation coefficients (*ρ*).

Factor	Ref country	Time interval	*n*	*θ*	*ρ*	95%CI	*p*-value	Signif
effd1	Mexico	2010-1 to 2015-40	34	2000	0.617	(0.352, 0.790)	1.034e-04	***
effd2	Mexico	2010-1 to 2015-40	34	2000	0.626	(0.365, 0.796)	7.509e-05	****
effd1	China	2010-1 to 2015-40	34	2000	−0.158	(−0.471, 0.191)	0.3733	
longitude	—	2010-1 to 2015-40	34	2000	0.538	(0.244, 0.741)	0.0010	**
latitude	—	2010-1 to 2015-40	34	2000	0.041	(−0.301, 0.374)	0.8173	
effd1	Mexico	2010-1 to 2016-8	34	2000	0.495	(0.188, 0.714)	0.0029	**
effd2	Mexico	2010-1 to 2016-8	34	2000	0.551	(0.261, 0.749)	7.423e-04	***
effd1	China	2010-1 to 2016-8	34	2000	−0.196	(−0.501, 0.152)	0.2662	
longitude	—	2010-1 to 2016-8	34	2000	0.537	(0.243, 0.741)	0.0011	**
latitude	—	2010-1 to 2016-8	34	2000	−0.071	(−0.400, 0.274)	0.6894	
effd1	Mexico	2006-1 to 2016-8	34	2000	0.483	(0.173, 0.706)	0.0038	**
effd2	Mexico	2006-1 to 2016-8	34	2000	0.537	(0.243, 0.741)	0.0011	**
effd1	China	2006-1 to 2016-8	34	2000	−0.215	(−0.516, 0.133)	0.2223	
longitude	—	2006-1 to 2016-8	34	2000	0.520	(0.221, 0.730)	0.0016	**
latitude	—	2006-1 to 2016-8	34	2000	−0.038	(−0.372, 0.304)	0.8297	
effd1	Mexico	2006-1 to 2008-52	30	2000	0.286	(−0.083, 0.586)	0.1252	
effd2	Mexico	2006-1 to 2008-52	30	2000	0.354	(−0.007, 0.634)	0.0548	*
effd1	China	2006-1 to 2008-52	30	2000	−0.156	(−0.489, 0.217)	0.4113	
longitude	—	2006-1 to 2008-52	30	2000	0.295	(−0.073, 0.593)	0.1130	
latitude	—	2006-1 to 2008-52	30	2000	0.136	(−0.236, 0.473)	0.4749	
effd1	Mexico	2006-1 to 2016-8	73	500	0.350	(0.130, 0.537)	0.0024	**
effd2	Mexico	2006-1 to 2016-8	73	500	0.411	(0.200, 0.586)	3.046e-04	***
effd1	China	2006-1 to 2016-8	73	500	−0.135	(−0.354, 0.098)	0.2551	
longitude	—	2006-1 to 2016-8	74	500	0.443	(0.239, 0.610)	7.767e-05	****
latitude	—	2006-1 to 2016-8	74	500	−0.099	(−0.321, 0.132)	0.3998	

*n*: number of countries in the study. *θ*: threshold of influenza specimens. For statistical significance levels, ‘^*^’ refers to *p*-value range of 0.01 but less than 0.1, ‘^**^’ refers to range of 0.001 but less than 0.01, ‘^***^’ refers to range of 0.0001 but less than 0.001, and ‘^****^’ refers to range of 0.00001 but less than 0.0001.

**Table 2:** Summary of all Pearson’s correlation coefficients for the US.

Time interval	*ρ*	95% CI	*p*-value	Signif
1997–2008 vs 2010–2015	−0.5208	(−0.88, 0.219)	0.1506	
1998–2008 vs 2010–2015	−0.5001	(−0.874, 0.246)	0.1704	
1999–2008 vs 2010–2015	−0.5814	(−0.899, 0.135)	0.1006	
2000–2008 vs 2010–2015	−0.6186	(−0.909, 0.077)	0.0757	
2001–2008 vs 2010–2015	−0.6289	(−0.912, 0.061)	0.0696	
2002–2008 vs 2010–2015	−0.7027	(−0.932, −0.072)	0.0347	*
2003–2008 vs 2010–2015	−0.7517	(−0.944, −0.175)	0.0195	*
2004–2008 vs 2010–2015	−0.7024	(−0.932, −0.072)	0.0349	*
2005–2008 vs 2010–2015	−0.7489	(−0.944, −0.169)	0.0202	*
2006–2008 vs 2010–2015	−0.6112	(−0.907, 0.089)	0.0804	
2007–2008 vs 2010–2015	−0.6498	(−0.918, 0.025)	0.0582	

*ρ* stands for correlation coefficient.
