# Supplementary material for: Spatio-temporal patterns of proportions of influenza B cases
Source: Sci Rep. 2017 Jan 9;7:40085. doi: 10.1038/srep40085 (PMC5220367; doi:10.1038/srep40085)
Supplement: Supplementary Information [file srep40085-s1.pdf]

Spatio-temporal patterns of proportions of influenza B cases

Daihai He[1], Alice PY Chiu[1], Qianying Lin[1] and Duo Yu [2]

[1] Department of Applied Mathematics, Hong Kong Polytechnic University

Hung Hom, Kowloon, Hong Kong (SAR) China

[2] Department of Biostatistics, School of Public Health

University of Texas Health Science Center at Houston, United States

November 12, 2016

Supplementary Material

Contents

S1 Effects of excluding data from 2010 and 2011 **2**

S2 Proportions of B virus v.s. country median age **2**

S3 Patterns of total confirmations **3**

S4 Patterns of proportions of B Lineages **5**

S4.1 Proportions of B Lineages v.s. median age 5

S4.2 Confirmations of B Lineages v.s. effective distances 6

S4.3 Proportions of B Lineages v.s. longitude and latitude 8

S5 A variety of models with or without region as a random-effect factor **10**

S1 Effects of excluding data from 2010 and 2011

Table S1: Effects of setting the start date to 2011-1 and 2012-1.

factor	ref country	time interval	n	θ	ρ	95%CI	p-value	signif
effd1	Mexico	2011-1 to 2015-40	31	2000	0.633	(0.359, 0.806)	1.324e-04	***
effd2	Mexico	2011-1 to 2015-40	31	2000	0.652	(0.388, 0.818)	6.957e-05	****
effd1	China	2011-1 to 2015-40	31	2000	-0.167	(-0.492, 0.199)	0.3683	
longitude	-	2011-1 to 2015-40	31	2000	0.558	(0.255, 0.762)	0.0011	**
latitude	-	2011-1 to 2015-40	31	2000	0.116	(-0.248, 0.452)	0.5341	
effd1	Mexico	2011-1 to 2016-8	31	2000	0.450	(0.114, 0.694)	0.0110	*
effd2	Mexico	2011-1 to 2016-8	31	2000	0.513	(0.194, 0.734)	0.0032	**
effd1	China	2011-1 to 2016-8	31	2000	-0.234	(-0.543, 0.131)	0.2046	
longitude	-	2011-1 to 2016-8	31	2000	0.533	(0.221, 0.747)	0.0020	**
latitude	-	2011-1 to 2016-8	31	2000	-0.018	(-0.370, 0.339)	0.9251	
effd1	Mexico	2012-1 to 2015-40	25	2000	0.563	(0.216, 0.784)	0.0034	**
effd2	Mexico	2012-1 to 2015-40	25	2000	0.599	(0.268, 0.804)	0.0015	**
effd1	China	2012-1 to 2015-40	25	2000	-0.320	(-0.635, 0.086)	0.1187	
longitude	-	2012-1 to 2015-40	25	2000	0.591	(0.256, 0.799)	0.0019	**
latitude	-	2012-1 to 2015-40	25	2000	0.087	(-0.319, 0.466)	0.6785	
effd1	Mexico	2012-1 to 2016-8	25	2000	0.412	(0.020, 0.694)	0.0408	*
effd2	Mexico	2012-1 to 2016-8	25	2000	0.447	(0.063, 0.716)	0.0250	*
effd1	China	2012-1 to 2016-8	25	2000	-0.313	(-0.630, 0.094)	0.1278	
longitude	-	2012-1 to 2016-8	25	2000	0.499	(0.129, 0.747)	0.0112	*
latitude	-	2012-1 to 2016-8	25	2000	-0.064	(-0.448, 0.340)	0.7611	

S2 Proportions of B virus v.s. country median age

Table S2: Summary of correlation tests of proportions of B virus against country median age. A marginally negative correlation is detected when a threshold of 500 cases is used.

factor	time interval	n	θ	ρ	95%CI	p-value	signif
median.age	2006-1 to 2016-8	74	500	-0.295	(-0.491, -0.072)	0.0106	*
median.age	2006-1 to 2008-52	48	500	-0.354	(-0.580, -0.078)	0.0135	*
median.age	2010-1 to 2016-8	74	500	-0.294	(-0.490, -0.071)	0.0109	*
median.age	2010-1 to 2015-40	74	500	-0.283	(-0.480, -0.058)	0.0147	*
median.age	2006-1 to 2016-8	34	2000	-0.025	(-0.361, 0.315)	0.8864	
median.age	2006-1 to 2008-52	30	2000	-0.260	(-0.567, 0.110)	0.1646	
median.age	2010-1 to 2016-8	34	2000	-0.054	(-0.385, 0.289)	0.7610	
median.age	2010-1 to 2015-40	34	2000	-0.120	(-0.440, 0.228)	0.5005	

We downloaded country median age from `https://en.wikipedia.org/wiki/List_of_countries_by_median_age` (checked on March 22, 2016).

S3 Patterns of total confirmations

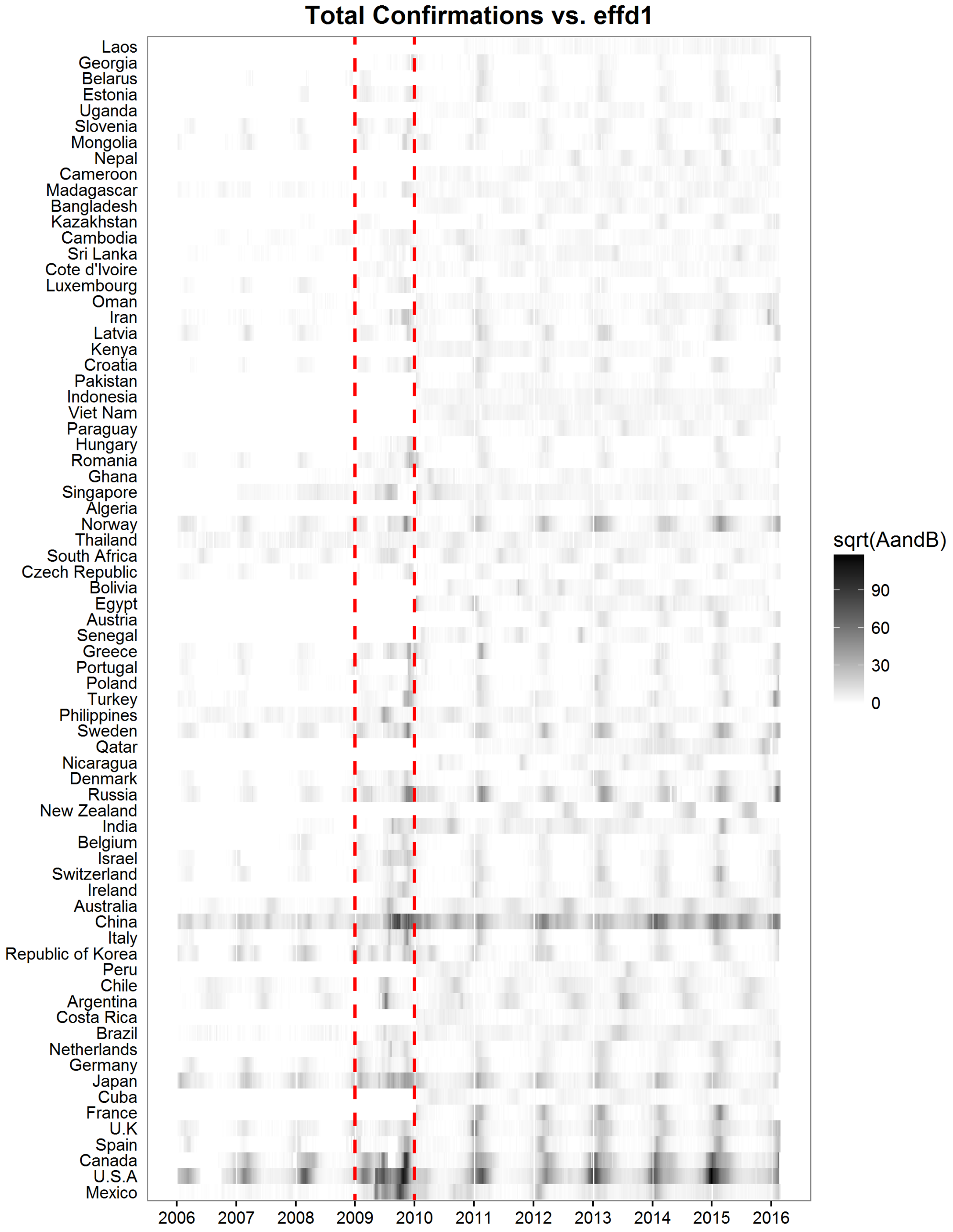

Figure S1: Patterns of total confirmations. Countries are ordered according to their effective distances to Mexico (definition 1).

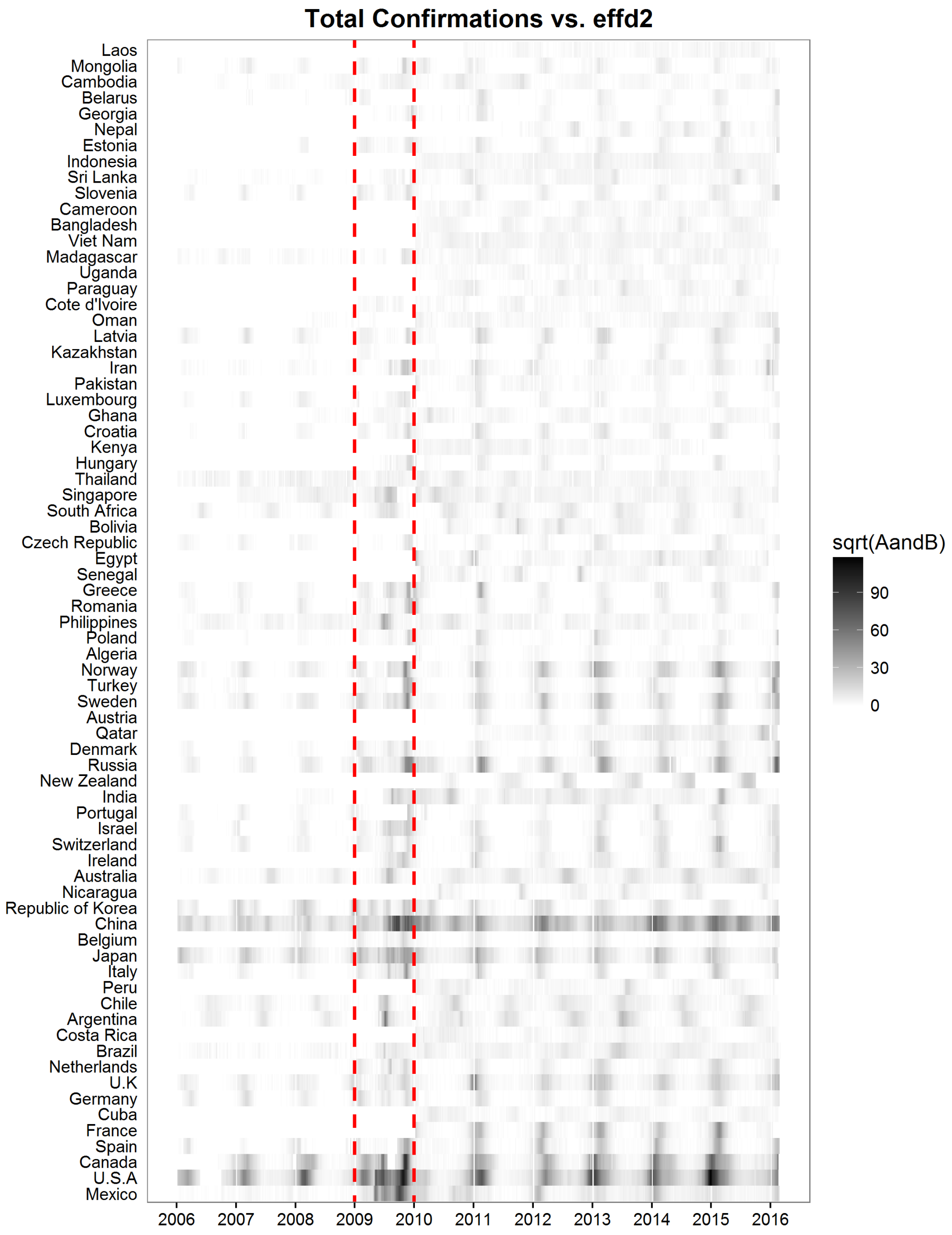

Figure S2: Patterns of total confirmations. Countries are ordered according to their effective distances to Mexico (definition 2).

S4 Patterns of proportions of B Lineages

S4.1 Proportions of B Lineages v.s. median age

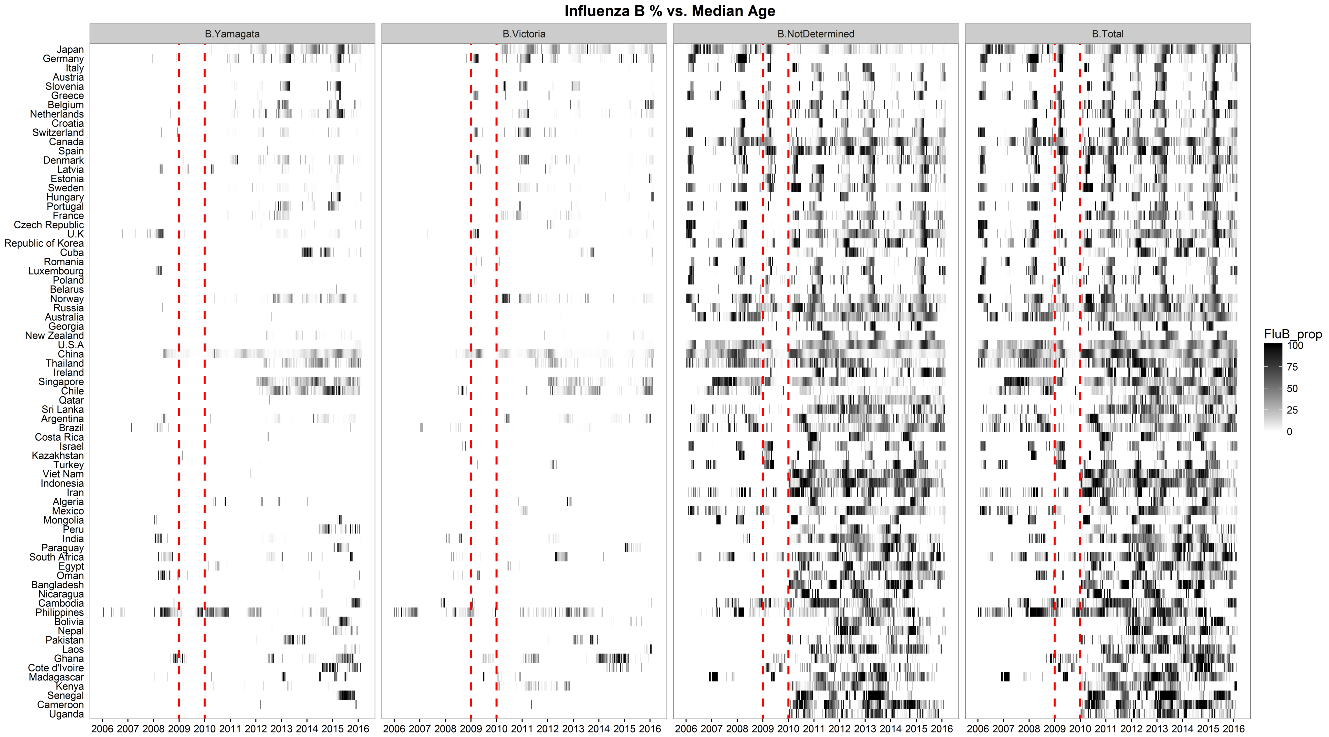

Figure S3: Patterns of proportions of different B lineages out of total of A and B. Countries are ordered according to the population's median ages.

S4.2 Confirmations of B Lineages v.s. effective distances

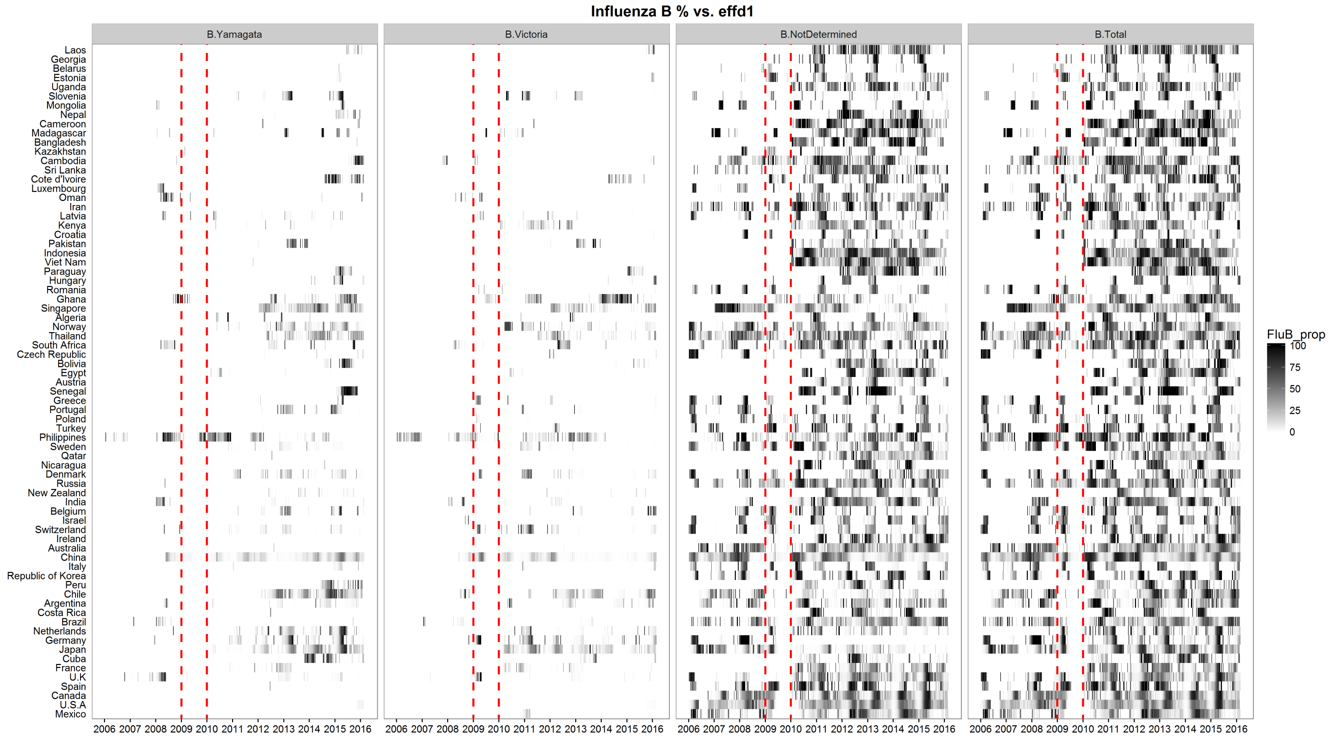

Figure S4: Patterns of proportions of different B lineages out of total of A and B. Countries are ordered according to the effective distance (definition 1) from Mexico.

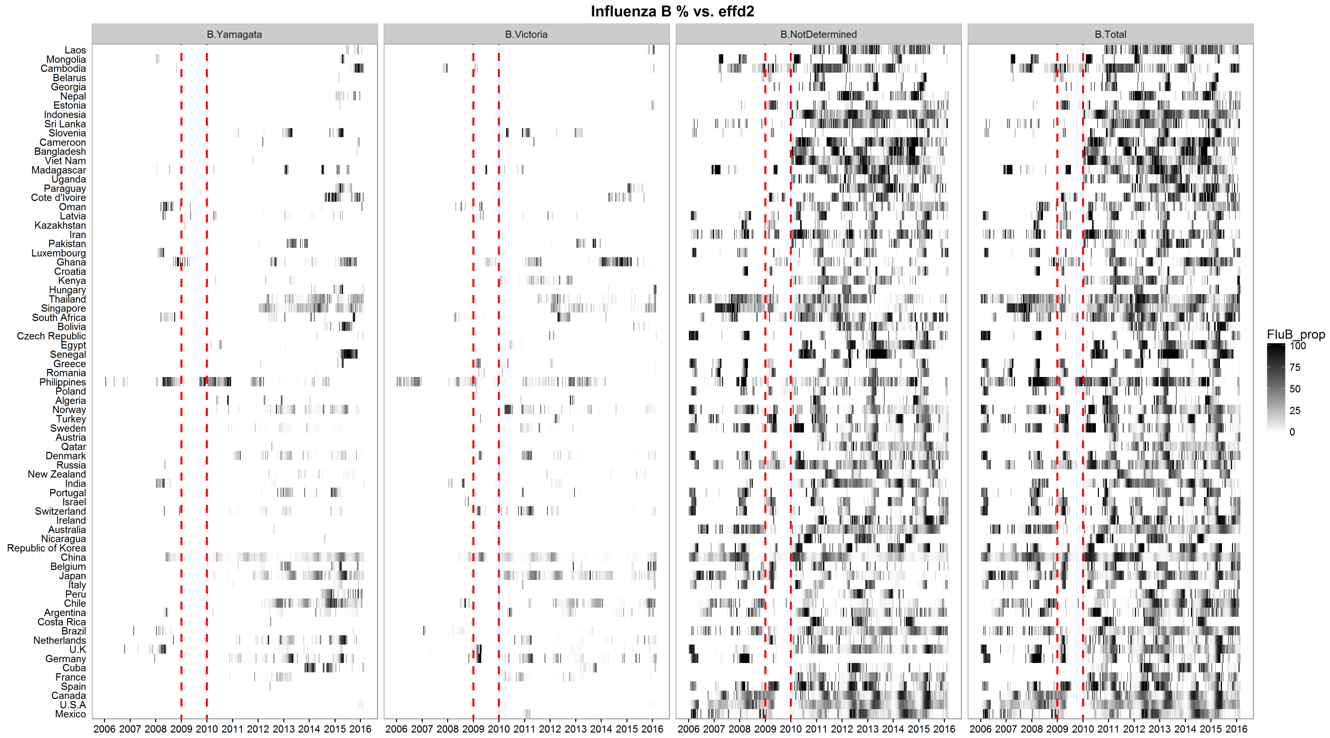

Figure S5: Patterns of proportions of different B lineages out of total of A and B. Countries are ordered according to the effective distance (definition 2) from Mexico.

S4.3 Proportions of B Lineages v.s. longitude and latitude

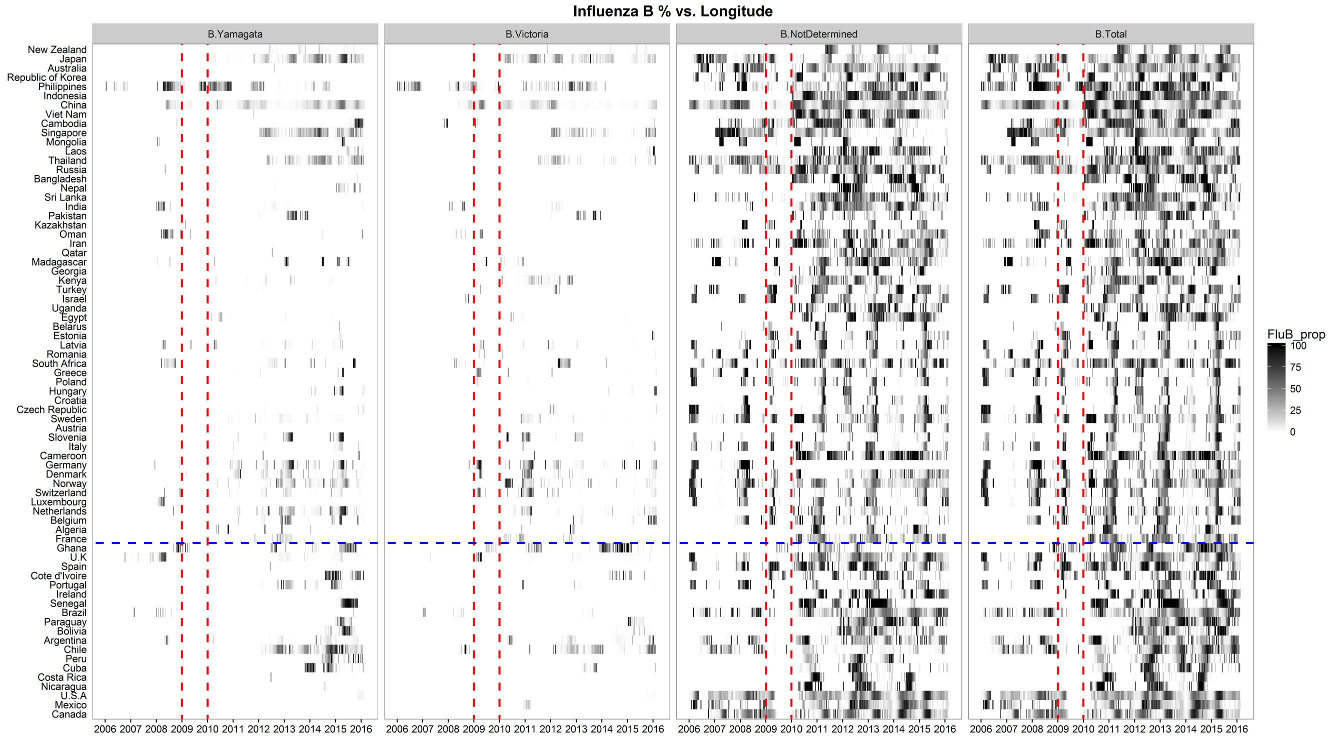

Figure S6: Patterns of proportions of different B lineages out of total of A and B. Countries are ordered according to longitude.

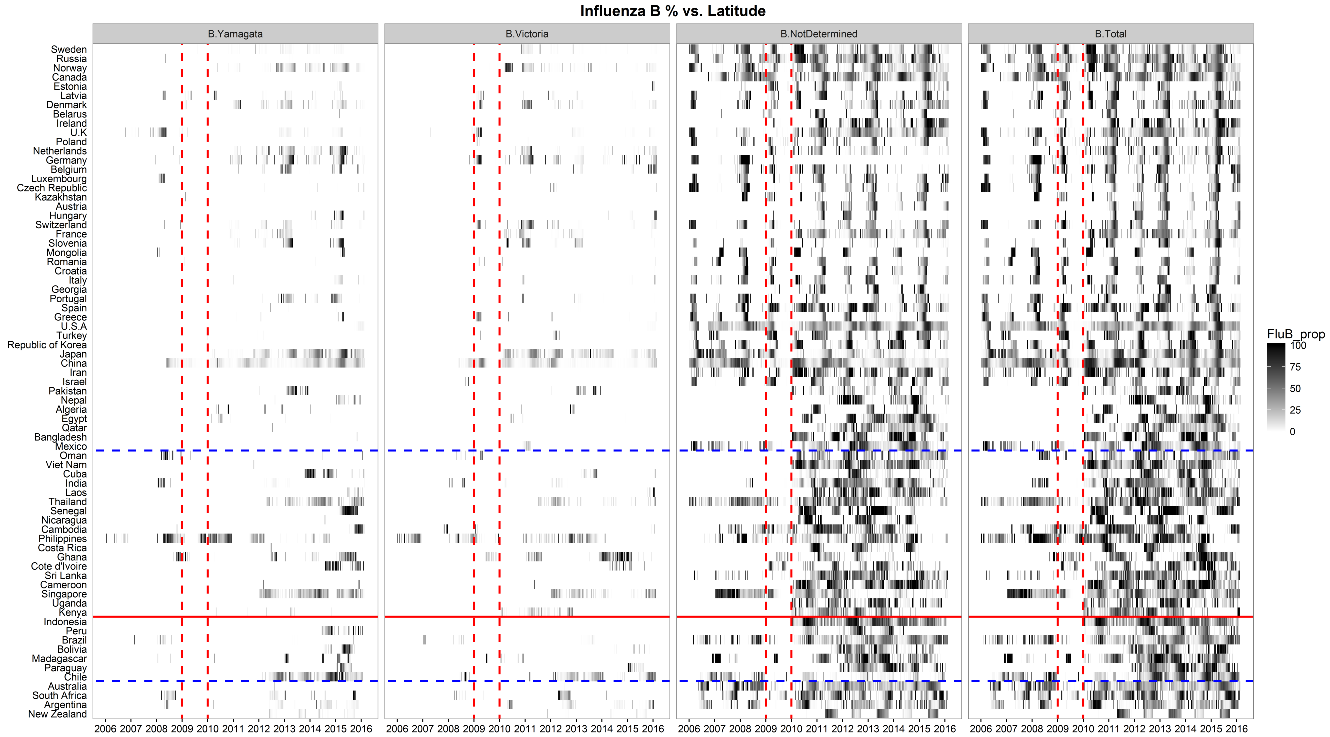

Figure S7: Patterns of proportions of different B lineages out of total of A and B. Countries are ordered according to latitude.

S5 A variety of models with or without region as a random-effect factor

```
A variety of models with or without region as a random-effect factor
Linear mixed model fit by REML ['lmerMod']
Formula: r ~ pop + long + abslat + effd + (1 | region)
   Data: rr

REML criterion at convergence: -162.7

Scaled residuals:
     Min       1Q   Median       3Q      Max
-2.41305 -0.52911  0.05728  0.69284  2.02150

Random effects:
 Groups   Name        Variance  Std.Dev.
 region   (Intercept) 0.0004447 0.02109
 Residual             0.0042312 0.06505
Number of obs: 77, groups:  region, 19

Fixed effects:
             Estimate Std. Error t value
(Intercept)  0.274755   0.009241  29.731
pop         -0.002384   0.008135  -0.293
long         0.022551   0.009811   2.299
abslat      -0.004060   0.008993  -0.451
effd         0.022670   0.009025   2.512

Correlation of Fixed Effects:
       (Intr) pop    long   abslat
pop    -0.008
long    0.019 -0.238
abslat  0.058 -0.009  0.050
effd    0.010  0.295 -0.418 -0.012
Linear mixed model fit by REML ['lmerMod']
Formula: r ~ specimens + long + abslat + effd + (1 | region)
   Data: rr

REML criterion at convergence: -161.5

Scaled residuals:
     Min       1Q   Median       3Q      Max
-2.41554 -0.48051 -0.00582  0.73237  2.03358
```

```
Random effects:
 Groups   Name        Variance  Std.Dev.
 region   (Intercept) 0.0006186 0.02487
 Residual             0.0039121 0.06255
Number of obs: 75, groups:  region, 19

Fixed effects:
             Estimate Std. Error t value
(Intercept)  0.277405   0.009716  28.550
specimens    0.007829   0.008800   0.890
long         0.027741   0.010224   2.713
abslat      -0.003936   0.009331  -0.422
effd         0.021356   0.009560   2.234

Correlation of Fixed Effects:
          (Intr) spcmns long   abslat
specimens -0.055
long       0.066  0.020
abslat     0.092 -0.126  0.073
effd      -0.045  0.381 -0.381 -0.075

Call:
lm(formula = r ~ pop + long + abslat + effd, data = rr)

Residuals:
      Min        1Q    Median        3Q       Max
-0.171101 -0.042221  0.005935  0.045810  0.153983

Coefficients:
             Estimate Std. Error t value Pr(>|t|)
(Intercept)  0.273880   0.007732  35.420  < 2e-16 ***
pop         -0.002944   0.008209  -0.359  0.72089
long         0.026653   0.008857   3.009  0.00361 **
abslat      -0.003699   0.007762  -0.477  0.63510
effd         0.020509   0.008941   2.294  0.02472 *
---
Signif. codes:  0 *** 0.001 ** 0.01 * 0.05 . 0.1   1

Residual standard error: 0.06785 on 72 degrees of freedom
  (1 observation deleted due to missingness)
Multiple R-squared:  0.2703,Adjusted R-squared:  0.2298
F-statistic: 6.668 on 4 and 72 DF,  p-value: 0.000127


Call:
```

```
lm(formula = r ~ specimens + long + abslat + effd, data = rr)

Residuals:
     Min       1Q   Median       3Q      Max 
-0.16828 -0.03902  0.00469  0.04140  0.15632 

Coefficients:
             Estimate Std. Error t value Pr(>|t|)    
(Intercept)  0.276333   0.007702  35.877  < 2e-16 ***
specimens    0.009057   0.008577   1.056 0.294610    
long         0.032161   0.008998   3.574 0.000641 ***
abslat      -0.004274   0.007685  -0.556 0.579894    
effd         0.020039   0.009602   2.087 0.040523 *  
---
Signif. codes:  0 *** 0.001 ** 0.01 * 0.05 . 0.1   1 

Residual standard error: 0.06656 on 70 degrees of freedom
  (3 observations deleted due to missingness)
Multiple R-squared:  0.3043,Adjusted R-squared:  0.2646 
F-statistic: 7.656 on 4 and 70 DF,  p-value: 3.552e-05 

Single term deletions

Model:
r ~ pop + long + abslat + effd + (region)
       Df Sum of Sq     RSS     AIC F value  Pr(>F)  
<none>              0.22646 -402.83                  
pop     1  0.001002 0.22747 -404.49  0.2388 0.62703  
long    1  0.006700 0.23316 -402.59  1.5977 0.21166  
abslat  1  0.000100 0.22656 -404.80  0.0238 0.87799  
effd    1  0.019464 0.24593 -398.48  4.6411 0.03569 *
region 18  0.104956 0.33142 -409.51  1.3904 0.17457  
---
Signif. codes:  0 *** 0.001 ** 0.01 * 0.05 . 0.1   1 
```
